# Data mining for health: staking out the ethical territory of digital phenotyping

**DOI:** 10.1038/s41746-018-0075-8

**Published:** 2018-12-19

**Authors:** Nicole Martinez-Martin, Thomas R. Insel, Paul Dagum, Henry T. Greely, Mildred K. Cho

**Affiliations:** 1 Stanford University, Palo Alto, CA USA; 2 Mindstrong Health, 248 Homer Street, Palo Alto, CA 94301 USA

**Keywords:** Health policy, Medical ethics

## Abstract

Digital phenotyping uses smartphone and wearable signals to measure cognition, mood, and behavior. This promising new approach has been developed as an objective, passive assessment tool for the diagnosis and treatment of mental illness. Digital phenotyping is currently used with informed consent in research studies but is expected to expand to broader uses in healthcare and direct-to-consumer applications. Digital phenotyping could involve the collection of massive amounts of individual data and potential creation of new categories of health and risk assessment data. Because existing ethical and regulatory frameworks for the provision of mental healthcare do not clearly apply to digital phenotyping, it is critical to consider its possible ethical, legal, and social implications. This paper addresses four major areas where guidelines and best practices will be helpful: transparency, informed consent, privacy, and accountability. It will be important to consider these issues early in the development of this new approach so that its promise is not limited by harmful effects or unintended consequences.

## Introduction

Digital phenotyping provides continuous, passive assessment of behavior, mood, and cognition by applying machine learning to physiological and biometric data gathered by smartphone and other personal digital devices.^1^ This paper focuses on the significant ethical concerns raised by digital phenotyping. While there are opportunities for digital phenotyping to assess a range of medical disorders, behavioral disorders have been the major focus thus far because of the urgent need for better measurement. Digital phenotyping technology involves the passive collection and mining of massive amounts of user data, transforming everyday actions into health information with the use of artificial intelligence, which is often not transparent or easily examined by outsiders. Given the attractiveness of digital phenotyping tools, it is not too early to consider the ethical, legal, and social implications of digital phenotyping in order to avoid unintended consequences. We will begin by defining digital phenotyping and its potential uses. In order to provide a foundation for implementing guidance for digital phenotyping, we identify key ethical considerations for implementation of the technology: accountability, protection of user data, transparency, and informed consent.

## What is digital phenotyping and how will it be used?

Broadly speaking, digital phenotyping refers to approaches in which personal data gathered from mobile devices and sensors is analyzed to provide health information. There is some variation in how the term has been defined. “Digital phenotyping” is sometimes more narrowly applied to the use of smartphones and wearables to collect data on physiological functions, such as pulse, or behavioral indicators, such as the user’s mobility, tapping and keyboard interactions, or features of voice or speech.^2–4^ Some approaches to digital phenotyping include the study of “digital exhaust,” such as social media posts and internet searches, as an indicator of health risks.^5–8^ In most current models of digital phenotyping, data collection is passive—once the wearable or app is downloaded, it collects information while the users otherwise go about their daily activities. Some forms of digital phenotyping, such as keyboard interactions, are “content-free,” meaning that only reaction times for tapping or scrolling are measured but the content of text or speech is not collected. Other forms of digital phenotyping which collect geolocation, search history, or social media posts can be described as “content-rich”.

Acquiring data is the first part of digital phenotyping. Analyzing these data to create insights about the user is the second part. Usually the algorithms developed to analyze this complex, multi-dimensional data are derived from some form of machine learning. These results are predictors of risk or probabilities, although they may be used for binary decisions (hospitalize vs discharge, alter medication vs continue status quo, etc.). Thus far, most published reports of digital phenotyping have focused on measures of mental health, such as relapse from depression or risk for psychotic episodes.^9–11^ Eventually, features associated with cognition such as executive function or verbal memory could be used to identify early signs of dementia,^12^ reduced alertness, risk of violent behavior, or predict severity of Parkinson’s disease.^13^

Digital phenotyping promises significant benefits when applied to medical uses. For psychiatry, which has heretofore relied exclusively on episodic reports of mood, digital phenotyping offers a powerful approach for the systematic detection of behavioral states,^14^ subtyping current heterogeneous diagnostic categories, and measuring outcomes. For neurology, which has required expensive, clinic-based assessments of cognitive performance, digital phenotyping offers an inexpensive, ecological assessment of function under real-world conditions. As digital phenotyping delivers rich data to both patients and providers, it may reconfigure the roles of both in the delivery of healthcare. The data analysis may also result in new insights that generate new categories for understanding mental disorder and risk.^14^

The ethical, legal, and social landscape will vary, depending on whether those with control over the data collection and the resulting data and analyses are medical researchers, clinicians, employers, educators, governments, consumers, or others (see Box 1). Some of the ethical concerns raised here, such as informed consent of patients who are children or have mental illness, are extensions of issues that arise with other digital health technologies, as well as in behavioral health as a whole. The novel ethical challenges posed by digital phenotyping arise from the way that the technology can transform seemingly mundane data into powerful indicators of mental function, with implications not only for healthcare but potentially in a range of areas where a measure of a change in cognitive performance or mood might have broad implications. For instance, within healthcare, digital phenotyping has the potential to gather and generate health-related information, such as a psychiatric diagnosis, outside the setting of a clinical encounter (i.e., through a direct-to-consumer app). Such use would be subject to regulations on informed consent and the Health Insurance Portability and Accountability Act (HIPAA). However, outside of healthcare, the regulatory frameworks are less clear.

Recent scandals involving Facebook and Cambridge Analytica are unfortunate reminders of the vulnerability of individuals to, and the relative ease of, the large-scale misuse of personally identifiable data that were detailed enough to create psychographic profiles of individuals.^15^ The military, employers, insurance organizations, and the criminal justice system could have interests in the prediction of behavioral states and disorders, as well as surveillance of individuals. The ability to collect and analyze data surreptitiously or to transform material that is voluntarily made available by individuals for their own purposes into data about those individuals’ psychological status raises novel issues of accountability and privacy. This technology will need to be designed and implemented so that it delivers benefits, while minimizing risks to individual users.

Box 1: Digital phenotyping usage The ethical, legal, and social landscape varies, depending upon the usage domain.

*Clinical Domain*: includes physician-mediated uses, as well as uses by hospitals and other healthcare providers as part of providing medical treatment patients, such as using voice acoustic features to predict severity of Parkinson’s disease. Data collected for these uses would generally be covered by HIPAA. This domain will foreseeably include hospitals or other healthcare institutions gathering data more broadly, raising questions of when and how patients might need to be informed of such uses.^46^

*Research Domain*: includes research performed by institutions with oversight by institutional review boards (IRBs) and covered by HIPAA, as well as research performed by industry developers that are potentially not covered by HIPAA. Apps, wearable sensors, and other digital technology pose challenges for IRB oversight, as IRBs may not be aware of potential risks posed by the technology to research subjects.^47^

*Government*: includes uses within the legal system, such as possible applications for criminal justice or civil commitment hearings, the military, or public health, such as the United Kingdom using digital phenotyping data to update alcohol consumption guidelines and interventions.^48^

*Education*: use by educational institutions to screen and monitor students for particular purposes. Examples of potential uses can be seen in University of Arizona using data from student identification cards to help identify those at risk of dropping out^49^ or colleges using mobile phone data to identify students at risk of depression or suicide.^50^

*Employment*: includes potential uses by employers to screen prospective employees, or for use in wellness programs.

*Consumer Domain*: broad category that includes direct-to-consumer uses, as well as uses by companies for purposes such as insurance^51^ or marketing. This domain is of pressing ethical concern because of the unclear, or lack of, lines of accountability and regulation to support consumer safety, privacy, and informed consent. Relevant examples:

- Facebook recently blocked insurers that had been using Facebook data to identify ”conscientious” drivers.^52^
- The Apple Watch 4 includes two digital health features to monitor atrial fibrillation and sudden falls. Apple participated in the FDA pre-certification pilot program for digital health technology and received FDA clearance for the Apple Watch 4’s health features.^53^ The Apple Watch 4 features have raised questions regarding transparency in the FDA process and potential risks to users in relation to privacy, surveillance, and false positives.^54^

## Accountability

To the extent that the technology falls outside existing ethical and regulatory frameworks, digital phenotyping may raise specific accountability issues. Accountability for safety and efficacy, normally assessed by government agencies, is not well developed for digital health technologies. Many digital phenotyping tools could be classified as medical devices and thus subject to regulation by the Federal Drug Administration (FDA), while some potential uses would likely be outside of the FDA domain. The FDA has faced challenges in determining how to effectively regulate the range of emerging digital health offerings.^16^ In particular, regulation of devices based on machine learning presents particular difficulties, because the reasons for particular results or findings may not be accessible for evaluation.^17^ The FDA has announced a Digital Health Program and a Pre-certification Program for manufacturers, which involves a shift from a product-based approach to a more process-based approach and does not address the issue of evaluating specific machine learning devices.^18,19^

While safety and efficacy are important, mechanisms to assure accountability for issues such as privacy and informed consent are necessary as well. The General Data Protection Regulation Act (GDPR) in the European Union (EU) provides an example of stricter regulation for protecting personal data, allowing consumers easier access and more control over their data and requiring companies to explain data use in clear terms.^20^ The US companies, and potentially academic and clinical researchers, need to comply with the GDPR when collecting personal data of individuals located in EU countries.^21^ Some companies, such as Microsoft, have indicated that they will extend some of the privacy protection practices associated with GDPR to all their customers.^22^ While there is nothing equivalent in the United States, the California Consumer Right to Privacy Act, passed in mid-2018, will confer many of the same protections as GDPR in California.^23^

Development and applications of digital phenotyping will span commercial, government, and healthcare domains. If digital phenotyping leads to mistakes, it may not be clear who—clinicians, institutions, manufacturers—will be accountable for the errors. It is also not yet clear which accountability frameworks for professional or fiduciary obligations of competence and judgment^16^ or ethical standards, such as the best interest standard, apply to digital analytic systems. Furthermore, while liability laws could address some failures in the safety or effectiveness of digital phenotyping software devices, accountability is not just, or even mainly, about liability risks.

Assessment and open communication regarding the duties and obligations of the different institutions and individuals involved in developing and implementing the technology are necessary. Should there be reporting requirements for “failures” of digital phenotyping? If so, what should be reported, to whom, and who should have access to the resulting information? Would a consumer app have the same obligation that a medical professional has for reporting an individual’s high suicidal risk? If digital phenotyping offers great benefits in managing mental illness but Medicaid does not cover it, some patients will not be treated justly. If it turns out that, in practice, use of digital phenotyping leads to biases in outcomes for specific populations, who is responsible for communicating and addressing those biases? Some of these obligations, such as data protection and reporting, may involve implementation through the design of the technology—necessitating open communication regarding which values should be prioritized in design and who in the process should hold which obligations.

## Protection of user data

Protection of user data is a particularly important issue for digital phenotyping, for several reasons. First, collected data are generated in contexts that people do not ordinarily associate with healthcare, or even might not be recognized as data (e.g., keystroke patterns on digital devices), and thus are not necessarily protected by existing standards, such as HIPAA, which is applied to information collected in explicit healthcare contexts. Second, these data sources may include text messages, emails, and location data that are highly granular, especially in combination. As a result, people may be unaware of the risks of identifiability. Finally, data protection is especially critical because of the sensitivity of behavioral and mental health diagnoses and predictions and their potential impacts on employment, insurance, litigation, or other contexts. In some commercial contexts, people may have lowered expectations of privacy or be willing to share some personal data in order to receive a perceived benefit.^24^ However, that willingness to share data may be dependent on context and the nature of the benefit.^25^ People may be significantly less willing to risk exposure of behavioral or physiological health information for consumer applications, as Facebook learned when it allowed marketers to target users based on analysis of their emotional states.^26^ The Facebook incident highlights the appeal of digital technology for predicting behavior to marketers and other institutions, as well as an enduring obligation to protect personal information, especially in this digital age.

One basic question regarding data management is which institutions, individuals, or users should store or have access to the raw data, the analytic system, and the reports generated by the system. Institutions using the analytic system and resulting data would need to meet relevant data security requirements, with adequate measures in place to protect the security of the data. The details of those measures, and who shall prescribe them and monitor compliance, will need explicit definition and should be included in the informed consent process. In order to protect users against unwanted intrusions into their personal data, there may be a need for guidelines to establish what kind of data may be gathered for certain types of uses. One potential solution is to draw a line between data that are free of semantic content, such as physiologic measures or keystroke patterns, versus data that include semantic content, such as text or speech. However, there is growing awareness that data labeled as content-free still may be used to draw inferences that reveal personal information.^27^ This points to a need for further empirical research to help discern ethically significant distinctions that can be made between these types of data. Software developers and providers using digital phenotyping will also need clarification on any associated obligations to disclose findings from the analysis, such as predictions of suicidal ideation or other violent behavior, as well as guidance for providing information requested in subpoenas or search warrants.

## Transparency

Transparency plays a key role in building trust in digital technology.^28^ In digital phenotyping, transparency requires clarity about *what* is collected, *how* it is collected, and *when* it is collected. The range of what can be measured, as noted above, includes classes of personal data that many people may not want to share, such as location, sleep cycle, or recordings of voice and speech. The nature of the information that should be communicated will vary according to user domain and profile. Developers of digital phenotyping tools and institutions using the technology will need to communicate how the technology works, the data that are being collected, as well as potential limitations. Researchers and clinicians will need to describe precisely to patients and research subjects what data they will and will not collect for digital phenotyping. In commercial and government domains, there will need to be consideration of when and how people must be informed of digital phenotyping analyses of their data for specific uses.

A related issue involves transparency for data analysis. Clinicians will need to have information regarding the effectiveness, as well as the limitations, of the software. Superficially, it may seem that patients and providers should have complete access to how digital data are translated into clinical insights. In practice, these algorithms are changing constantly as the system learns from newer data. While the overall analytic approach can and should be described so that there is transparency about the method, the specifics of which items, with specific weights, contribute to a given risk estimate are likely to be evanescent and therefore misleading. Furthermore, algorithms developed within private sector companies are generally protected as intellectual property and, therefore, not fully transparent. This has proven problematic in other areas, such as when algorithms used for predicting recidivism were criticized for racial bias.^29^ In the consumer or government domains,individuals could face repercussions from findings informed by artificial intelligence (AI) (e.g., sentencingdeterminations or adjustments to government benefits) yet not be able to examine and thereby challengethe reasoning behind those findings. One solution that has been proposed to this problem is developing AI systems that can “explain” their results.^30^ Finally, machine learning or other approaches used to predict risk or to identify probabilities will inevitably create false positives and false negatives. There are many potential uses, such as in criminal justice, that would involve applying digital phenotyping risk probabilities to binary decisions. It will be necessary to provide relevant training or informational material, tailored to the particular use, whether clinical, government, or consumer, to understand the nature and limitations of digital phenotyping predictions for the specific application.^31^

## Informed consent

The collection of digital data is ostensibly of relatively low risk, as it consists of the same activities an individual would otherwise engage in. However, digital phenotyping and its consequences, intended and otherwise, are new and largely unknown to patients or others who may be subject to it. Individuals will need to understand when and by whom their data are collected, where their data will be stored, who will have access to these data, and how their data could be used, including the types of inferences that could and will be made from them, and the magnitude of likelihood of inferential error.

Historically, the concept of informed consent is rooted in a construct in which primary control over medical information and resources rests in healthcare institutions and professionals.^32^ Digital phenotyping, in keeping with prior trends in digital and consumer health, shifts more responsibility for health information away from healthcare professionals to other actors, such as patients and consumers. The types of disclosure necessary to fulfill informed consent may vary across different domains and applications of digital phenotyping. There is increasing awareness of the need to inform consumers of potential consequences of how their data are used.^33^ In medical and research settings, informed consent generally requires full disclosure of relevant information, adequate comprehension, and voluntary choice.^34^ Across mental health applications of digital phenotyping, comprehension and voluntariness are challenges that must not be overlooked.

In healthcare, the consent process will need to explicitly define these data collection issues, as well as how the data will be used to inform medical decisions (including decisions about medication and hospitalization). If digital phenotyping data will be entered into the patient’s electronic health record (EHR), there may be a need to inform the patient of potential third-party access to the EHR.^35^ In non-medical settings, there need to be mechanisms to ensure that individuals are informed and give consent when their personal data are being collected and analyzed to generate health indicators. Many consumer apps use dense “terms and conditions” to convey information regarding obligations, risks, and benefits. Digital phenotyping technology should be configured to present these issues clearly to users for the purposes of consent. One potential solution is to take advantage of the technology to improve consent, such as staging the disclosures to highlight key information.^36^ Regulation also can help set forth what and how information must be discussed. For example, under the GDPR, consumers must be informed in concise and plain language how their data are being collected and used.^37^ Such efforts might also need to address more subtle business practices that can have coercive effects on consumers, such as requiring data collection in return for access to certain services or making it difficult to find the privacy settings.^38,39^

Children and individuals with severe mental illness raise special informed consent considerations. Youth are among the heaviest users of smartphones.^40^ With many mental disorders beginning before adulthood, this may be the demographic most likely to benefit from early detection of depression or psychosis. Issues such as parental consent for a child, or whether teens can consent, need to be considered carefully. Furthermore, the issue of when to obtain consent can become complicated. People who agreed to the monitoring necessary for digital phenotyping while symptoms have abated may be more upset about being monitored when they are experiencing symptoms such as delusions, anxiety, or psychosis. In healthcare settings, one approach to this situation is to inform patients of their agency in this process, and that they can terminate monitoring when they wish. At the same time, the consequences of terminating the app or the wearable must be sufficiently set forth, such as whether termination would result in reduced care, increased costs, or alteration of the clinician–patient relationship.

Currently, digital phenotyping is limited to clinical studies with identified patients or research volunteers who give consent individually. If this approach proves useful for monitoring cognitive performance or risks for psychological distress, will it be deployed broadly in work or school environments? There would then need to be attention to what kinds of disclosure and consent are necessary in these settings to maintain trust and transparency. In employment or military settings, there will need to be attention to coercive practices that could undermine the voluntary nature of consent. The ease of collecting these kind of data lends itself to scale, potentially to millions of people outside of clinical care. Digital phenotyping is already being proposed for use in monitoring signs of depression in undergraduates, identifying suicide risk early^41^ or defining risk groups in an adult population for life insurance eligibility. It is not too early to consider how and when consent should be obtained under such circumstances. Absent this careful consideration, an approach that was developed for medical management could become a tool for population surveillance.

## Conclusion

Digital phenotyping could revolutionize how we measure cognition, mood, and behavior. Currently, this technology is being validated in carefully controlled, large-scale trials.

Because digital phenotyping uses a ubiquitous technology and is inexpensive to deploy, it will likely transform the diagnosis and treatment of mental illness globally by enabling passive, continuous, quantitative, and ecological measurement-based care. As with any promising new approach, the risks and unintended consequences need to be considered to ensure the safe and trusted development of digital phenotyping. Direct-to-consumer applications raise particular concerns regarding data protection that may be productively addressed through regulation, as well as development of industry standards. As digital phenotyping moves forward, researchers will benefit from developing a conceptual framework on which to base standards for the collection, processing, and reporting of digital phenotyping data. Collaborative efforts between developers and researchers, as occurred in the field of genetics, will be necessary for developing these standards.^42^

Because existing ethical and regulatory frameworks for the provision of mental healthcare do not clearly apply to digital phenotyping, stakeholders, including software developers, healthcare, patients, consumers, and other institutions will need to be involved in the creation of standards and best practices that adequately address the ethical challenges raised here. Empirical research will be needed to better understand the nature and scope of some of these ethical challenges, such as how clinicians and patients understand and act upon digital phenotyping findings. There are already some efforts underway to address ethical issues raised by digital health, such as the National Institute of Mental Health (NIMH) task force to address the use of informational technologies for mental health.^43–45^ Digital phenotyping involves ethical challenges across different institutional domains, and hence collaborative efforts across relevant disciplines and stakeholders are especially needed.
